# Anti-Inflammatory Effect of Combination of Scutellariae Radix and Liriopis Tuber Water Extract

**DOI:** 10.1155/2015/203965

**Published:** 2015-10-29

**Authors:** Mi-Hye So, You-Kyung Choi

**Affiliations:** Department of Korean International Medicine, College of Korean Medicine, Gachon University, 1342 Seongnamdaero Sujeong-gu, Seongnam-si, Gyeonggi-do, Republic of Korea

## Abstract

Scutellariae Radix and Liriopis Tuber have been used to treat the inflammatory diseases in traditional Korean medicine and anti-inflammatory effect of each herb has been shown partially in several articles. However, the combined extract of these medicinal herbs (SL) has not been reported for its anti-inflammatory effects. In this study, we investigated the effects of SL on the creation of several proinflammatory mediators in RAW 264.7 cell mouse macrophages induced by Lipopolysaccharide (LPS). SL inhibited significantly the increase of NO, the release of intracellular calcium, the increase of interleukin-6 (IL-6), macrophage inflammatory proteins (MIP-1*α*, MIP-1*β*, and MIP-2), and granulocyte colony-stimulating factor (G-CSF) in LPS-induced RAW 264.7 cell at the concentrations of 25, 50, and 100 *μ*g/mL, and SL inhibited significantly the increase of macrophage colony-stimulating factor (M-CSF) at the concentrations of 25 and 50 *μ*g/mL, and tumor necrosis factor (TNF) at the concentration of 25 *μ*g/mL. These results implicate that SL has anti-inflammatory effects by suppressing the production of various inflammatory mediators in macrophages. But SL did not inhibit significantly the increase of granulocyte macrophage colony-stimulating factor (GM-CSF), leukemia inhibitory factor (LIF), and Regulated on Activation, Normal T cell Expressed and Secreted (RANTES); therefore, further study is demanded for the follow-up research to find out the possibility of SL as a preventive and therapeutic medicine for various inflammatory diseases.

## 1. Introduction

Inflammation, a defense mechanism against various stimuli inducing injury, accompanies symptoms of flare, fever, swelling, pain, and various functional disorders. Many cytokines and proteins as well as prostaglandin E_2_ (PGE_2_), lysosomal enzymes, and free radicals are known to be involved in inflammation [1]. Macrophages, involving innate immunity via phagocytic process as well as acquired immunity via antigen-presenting function, are representative immune cells activated by pathogens, cellular injury, and cytokines secreted by different types of immune cells. The activated macrophages trigger inflammatory reactions by secreting cytokines, such as TNF-*α*, IL-1, and other various inflammatory mediators, that is, nitric oxide (NO), reactive oxygen species (ROS), and prostaglandins [2]. Lipopolysaccharide (LPS), a major outer membrane component of Gram-negative bacteria [3], acting as an external immunostimulator, known to generate various immune cells and to promote the secretion of proinflammatory cytokines, is an established model molecule for inflammation research [4]. Inflammation not just affects simple inflammatory reactions but also acts on inflammatory mediators, which mediate many chronic diseases and inflammation-related disorders, hence getting more attention. Anti-inflammatory medicines show their effectiveness by suppressing the metabolism of these inflammatory mediators; therefore, a lot of researches to discover novel candidates to control inflammatory reactions by regulating various chemical inflammatory mediators have been actively pursued [5].

According to a report, while many experiments employing the extracts of a single medicinal herb focused on in vitro effect, combined extracts have been rather tested for either animal or clinical researches [6]. Scutellariae Radix (SR) and Liriopis Tuber (LT) have shown their anti-inflammatory effects with their extract alone in several research articles [7–11], yet the combined extract of these medicinal herbs has not been reported for its anti-inflammatory effect.

In this study, anti-inflammatory effect of the mixture of Scutellariae Radix (SR) and Liriopis Tuber (LT) was evaluated. Each plant was extracted alone in hot water and then combined mixture called sample “SL” was tested in RAW 264.7, a mouse macrophage cell line, for cell viability rate and the effect of NO production. The levels of NO production, intracellular free calcium, and production of several cytokines, such as interleukin (IL), macrophage inflammatory protein (MIP), granulocyte colony-stimulating factor (G-CSF), macrophage colony-stimulating factor (M-CSF), granulocyte macrophage colony-stimulating factor (GM-CSF), tumor necrosis factor (TNF), leukemia inhibitory factor (LIF), and Regulated on Activation, Normal T cell Expressed and Secreted (RANTES), were significantly affected by the treatment of the sample “SL” in RAW 264.7 cells stimulated by LPS.

## 2. Materials and Methods

### 2.1. Preparation of Sample (SL)

20 g of Scutellariae Radix and 20 g of Liriopis Tuber were extracted in 2,000 mL of distilled water each using a reflux extractor. The decoction was boiled for 2 hrs after temperature reached boiling point. Each extract was vacuum-filtered through filter papers (Advantec No. 2, Japan) and then concentrated in a rotary vacuum evaporator. Each concentrate was freeze-dried, resulting in 6.9 g of Scutellariae Radix (extraction yield, 34.5%) and 4.5 g of Liriopis Tuber (extraction yield, 22.5%). The mixed extracts (1 : 1 ratio) yielded the testing material (SL), which was used throughout in this study.

### 2.2. Cell Culture

RAW 264.7 cells were cultured in DMEM medium with 10% FBS, penicillin (100 U/mL), and streptomycin (100 *μ*g/mL) at 37°C under 5% CO_2_ in a CO_2_ culture incubator. The cells were sufficiently expanded in 75 cm^2^ flask (Falcon, USA), washed with phosphate buffered saline (PBS) every 3 days. The cells were then treated with 0.25% trypsin-EDTA (1 mL/50 mL flask) for 1 min and incubated at 37°C for additional 5 min to detach from the culture flask after trypsin solution was removed. The resulting detached cells suspended in 10 mL DMEM 10% FBS were split 1 : 2 ratio and divided into new culture flasks and incubated in the CO_2_ incubator (37°C, 5% CO_2_) [7].

### 2.3. Cell Viability Assay

MTT assay was carried out as follows [12, 13]. 100 *μ*L of the cells (1 × 10^5^ cells/well) was added into a 96-well plate and incubated at 37°C, 5% CO_2_ incubator for 24 hrs. After the plate was washed with phosphate buffered saline (1x PBS), various concentrations of the sample in PBS of the same volume were treated in the designated wells and incubated. At the end of incubation, 100 *μ*L of 1 mg/mL MTT (Sigma, USA) in PBS was added to each well. The plate was protected from light with aluminum foil wrapping and incubated at the same condition for 2 hrs. After removing the culture medium, 100 *μ*L DMSO was added to the wells and left at 37°C for 2 hrs. Cell viability was assessed by measuring the absorption in a microplate reader (Molecular Devices, USA) at 490 nm.

### 2.4. Measurement of NO Production

L-Arginine, the substrate of NO, is converted to L-citrulline and nitric oxide (NO), which are quickly stabilized to NO_2_, nitrite, and nitrate. Griess reagent (0.5% sulfanilamide, 2.5% phosphoric acid, and 0.5% naphthylethylenediamine) chemically reacts with nitrite, forming azo dye in purple color, of which concentration is identical to that of NO. The nitrite concentration was estimated from azo dye concentration; therefore, the microplate reader was set to measure the absorption at 540 nm to determine the production of NO. To investigate the effect of the testing sample (SL) on the production of NO in RAW 264.7 cells, the following experiments were carried out. LPS (1 mg/mL) alone or with various concentrations of the testing sample (SL) was treated to each well and incubated at 37°C, 5% CO_2_ incubator for 24 hrs. The supernatant (100 *μ*L) added to a 96-well plate was mixed with 100 *μ*L Griess reagent and reacted for 15 min. The NO production was estimated by measuring the absorption at 540 nm with a microplate reader (Bio-Rad, USA) [14].

### 2.5. Intracellular Free Calcium Measurement

To investigate the effect of the testing sample (SL) on the intracellular free calcium release in RAW 264.7 cells, fluo-4 calcium assay was carried out as follows. 100 *μ*L of the cells (1 × 10^5^ cells/well) was added into a 96-well plate and incubated at 37°C, 5% CO_2_ incubator for 24 hrs. LPS (1 *μ*g/mL) alone or with various concentrations of the testing sample (SL) was treated to each well and incubated at 37°C, 5% CO_2_ incubator for 18 hrs. After the plate was washed with phosphate buffered saline (1x PBS), various concentrations of the sample in PBS of the same volume were treated in the designated wells and incubated. After the incubation, the medium in the wells was removed and 100 *μ*L fluo-4 dye solution was added to each well and incubated at 37°C, 5% CO_2_ incubator for 30 min. The fluorescence intensity of each well was measured by spectrofluorometer (485 nm for excitation filter; 535 nm for emission filter) [14].

### 2.6. Measurement of Cytokine Level

The following experiment by referencing Ryu et al. [10, 14, 15] was carried out to find whether the testing sample (SL) affects the secretion of immunoproteins. 100 *μ*L of the cells (1 × 10^5^ cells/well) was added into a 96-well plate and incubated at 37°C, 5% CO_2_ incubator for 24 hrs. After the plate was washed with phosphate buffered saline (1x PBS), various concentrations of the sample in PBS in the same volume of the medium were treated in the designated wells and incubated. LPS (1 *μ*g/mL) alone or with various concentrations of the testing sample (SL) was treated to each well and incubated at 37°C, 5% CO_2_ incubator. At the end of incubation, cell culture supernatant from each well was reacted with the antibody-conjugated capture beads put in the wells in advance. The reacted capture beads in the wells of a filter plate were washed in 150 *μ*L per well of the washing buffer. After reacting with added detection antibody, the plate was incubated for 30 min. After three times of washing in the washing buffer, 120 *μ*L reading buffer was added into each well, and 5 min of shaker culture (300~500 rpm) at the room temperature was followed. Using Bio-Plex array reader (Bio-Plex 200), the target cytokine levels were determined.

### 2.7. Statistical Analysis

All the results in this study are expressed as mean ± SD, and the difference between control value and that of the test material was analyzed by Student's *t*-test. Less than 0.05 of *P* value was considered as statistically significant.

## 3. Results

### 3.1. The Effect to RAW 264.7 on the Cell Viability

**Figure 1:**
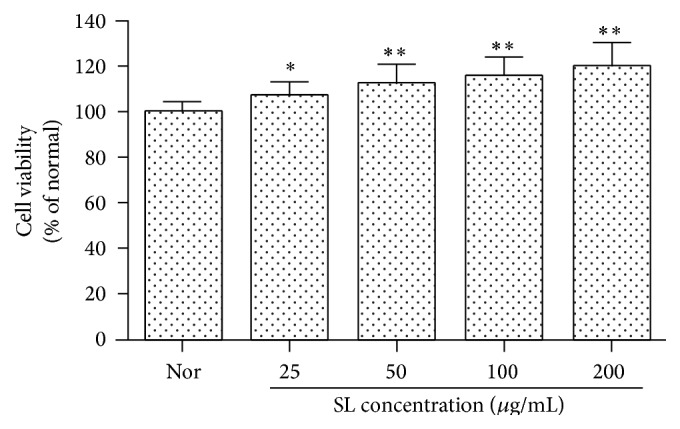
Effect of SL on cell viability in RAW 264.7 cells. Normal (Nor): treated with media only. SL: Scutellariae Radix and Liriopis Tuber water extract. Each RAW 264.7 cell group was incubated with SL at the concentrations of 25, 50, 100, and 200 *μ*g/mL for 24 hrs. The test material at the concentrations of 25, 50, 100, and 200 *μ*g/mL significantly increased the cell viability. Results are represented as mean ± SD. *∗* represents *P* < 0.05 compared to the normal. *∗∗* represents *P* < 0.01 compared to the normal.

The test material at the concentrations of 25, 50, 100, and 200 *μ*g/mL significantly increased the cell viability (Figure 1).

### 3.2. The Effect on the NO Production

#### 3.2.1. In the Simple RAW 264.7 Cells

**Figure 2:**
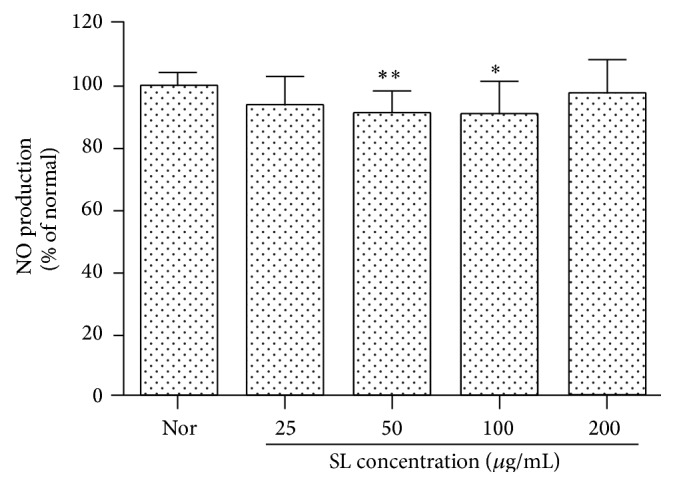
Effect of SL on NO production in RAW 264.7 cells. Normal (Nor): treated with media only. SL: Scutellariae Radix and Liriopis Tuber water extract. Each RAW 264.7 cell group was incubated with SL at the concentrations of 25, 50, 100, and 200 *μ*g/mL for 24 hrs. SL at the concentrations of 50 and 100 *μ*g/mL inhibited the production of NO significantly. Results are represented as mean ± SD. *∗* represents *P* < 0.05 compared to the normal. *∗∗* represents *P* < 0.01 compared to the normal.

The result of 24-hour treatment of SL to RAW 264.7 cells showed that SL at the concentrations of 50 and 100 *μ*g/mL inhibited the production of NO significantly (Figure 2).

#### 3.2.2. In the LPS-Stimulated RAW 264.7 Cells

**Figure 3:**
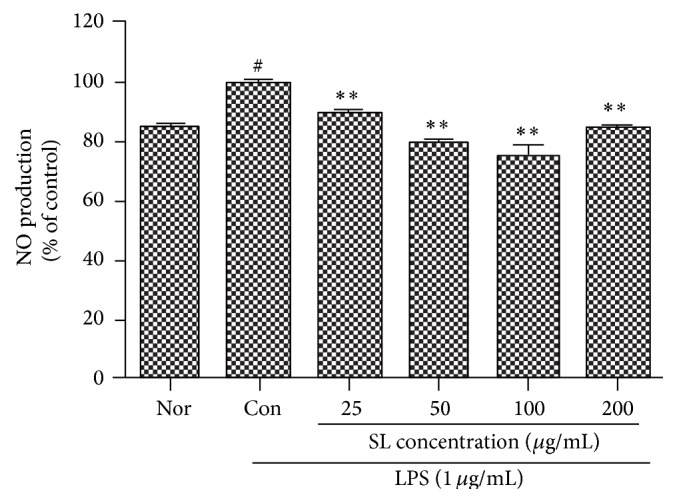
Effect of SL on NO production in LPS-treated RAW 264.7 cells. Normal (Nor): treated with media only. Control (Con): treated with LPS (1 *μ*g/mL). SL: Scutellariae Radix and Liriopis Tuber water extract. Each RAW 264.7 cell group was incubated with SL at the concentrations of 25, 50, 100, and 200 *μ*g/mL with LPS for 24 hrs. The production of NO in the LPS-stimulated RAW 264.7 cells was decreased significantly at all the concentrations of SL (25, 50, 100, and 200 *μ*g/mL). Results are represented as mean ± SD. # represents *P* < 0.05 compared to the normal. *∗∗* represents *P* < 0.01 compared to the control.

When RAW 264.7 cells were cotreated with LPS (1 *μ*g/mL) and various concentrations of SL for 24 hrs, the production of NO in the LPS-stimulated RAW 264.7 cells was decreased significantly at all the concentrations of SL (25, 50, 100, and 200 *μ*g/mL) (Figure 3).

### 3.3. The Effect on the Intracellular Free Calcium in the LPS-Stimulated RAW 264.7 Cells

**Figure 4:**
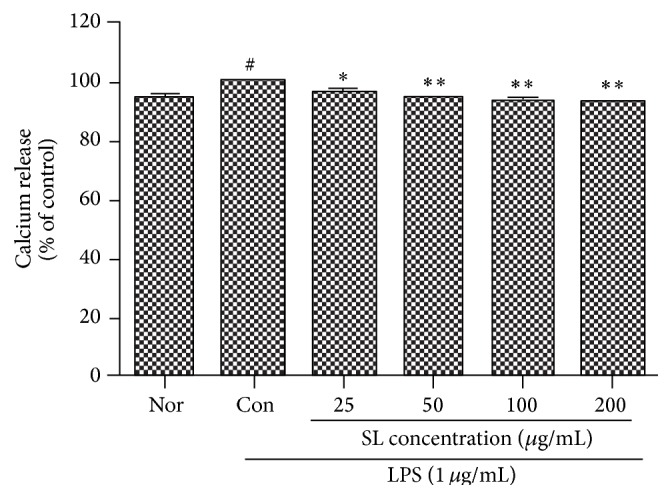
Effect of SL on calcium release in LPS-treated RAW 264.7 cells. Control (Con): treated with LPS (1 *μ*g/mL). SL: Scutellariae Radix and Liriopis Tuber water extract. Each RAW 264.7 cell group was incubated with SL at the concentrations of 25, 50, 100, and 200 *μ*g/mL with LPS for 18 hrs. The increase of intracellular free calcium in the LPS-stimulated RAW 264.7 cells was inhibited significantly by the treatment of SL at all the concentrations (25, 50, 100, and 200 *μ*g/mL). Results are represented as mean ± SD. # represents *P* < 0.05 compared to the normal. *∗* represents *P* < 0.05 compared to the control. *∗∗* represents *P* < 0.01 compared to the control.

When RAW 264.7 cells were cotreated with LPS (1 *μ*g/mL) and various concentrations of SL for 18 hrs, the increase of intracellular free calcium in the LPS-stimulated RAW 264.7 cells was inhibited significantly by the treatment of SL at all the concentrations (25, 50, 100, and 200 *μ*g/mL) (Figure 4).

### 3.4. The Effects on Cytokines Production

#### 3.4.1. IL-6 Production in RAW 264.7 Cells

When RAW 264.7 cells were cotreated with LPS (1 *μ*g/mL) and various concentrations of SL for 24 hrs, the increase of the production of IL-6 in the LPS-stimulated RAW 264.7 cells was significantly inhibited by the treatment of SL at all the concentrations (25, 50, and 100 *μ*g/mL) (Table 1).

#### 3.4.2. MIP-1a Production in RAW 264.7 Cells

When RAW 264.7 cells were cotreated with LPS (1 *μ*g/mL) and various concentrations of SL for 24 hrs, the increase of the production of MIP-1a in the LPS-stimulated RAW 264.7 cells was significantly inhibited by the treatment of SL at all the concentrations (25, 50, and 100 *μ*g/mL) (Table 1).

#### 3.4.3. MIP-1*β* Production in RAW 264.7 Cells

When RAW 264.7 cells were cotreated with LPS (1 *μ*g/mL) and various concentrations of SL for 24 hrs, the increase of the production of MIP-1*β* in the LPS-stimulated RAW 264.7 cells was significantly inhibited by the treatment of SL at all the concentrations (25, 50, and 100 *μ*g/mL) (Table 1).

#### 3.4.4. MIP-2 Production in RAW 264.7 Cells

When RAW 264.7 cells were cotreated with LPS (1 *μ*g/mL) and various concentrations of SL for 24 hrs, the increase of the production of MIP-2 in the LPS-stimulated RAW 264.7 cells was significantly inhibited by the treatment of SL at all the concentrations (25, 50, and 100 *μ*g/mL) (Table 1).

#### 3.4.5. G-CSF Production in RAW 264.7 Cells

When RAW 264.7 cells were cotreated with LPS (1 *μ*g/mL) and various concentrations of SL for 24 hrs, the increase of the synthesis of G-CSF in the LPS-stimulated RAW 264.7 cells was significantly inhibited by the treatment of SL at all the concentrations (25, 50, and 100 *μ*g/mL) (Table 1).

#### 3.4.6. The Effect on M-CSF Production in RAW 264.7 Cells

When RAW 264.7 cells were cotreated with LPS (1 *μ*g/mL) and various concentrations of SL for 24 hrs, the increase of the synthesis of M-CSF in the LPS-stimulated RAW 264.7 cells was inhibited by the treatment of SL but not significantly (Table 1).

#### 3.4.7. The Effect on GM-CSF Production in RAW 264.7 Cells

When RAW 264.7 cells were cotreated with LPS (1 *μ*g/mL) and various concentrations of SL for 24 hrs, the increase of the synthesis of GM-CSF in the LPS-stimulated RAW 264.7 cells was inhibited by all the concentrations of treated SL but not significantly (Table 1).

#### 3.4.8. TNF-*α* Production in RAW 264.7 Cells

When RAW 264.7 cells were cotreated with LPS (1 *μ*g/mL) and various concentrations of SL for 24 hrs, the increase of the production of TNF-*α* in the LPS-stimulated RAW 264.7 cells was significantly inhibited by the treatment of SL at the concentration of 25 *μ*g/mL (Table 1).

#### 3.4.9. The Effect on LIF Production in RAW 264.7 Cells

When RAW 264.7 cells were cotreated with LPS (1 *μ*g/mL) and various concentrations of SL for 24 hrs, the increase of the synthesis of LIF in the LPS-stimulated RAW 264.7 cells was inhibited by the treatment of SL but not significantly (Table 1).

#### 3.4.10. The Effect on RANTES Production in RAW 264.7 Cells

When RAW 264.7 cells were cotreated with LPS (1 *μ*g/mL) and various concentrations of SL for 24 hrs, the increase of the synthesis of RANTES in the LPS-stimulated RAW 264.7 cells was inhibited by the treatment of SL but not significantly (Table 1).

## 4. Discussion

Inflammation is fundamentally an immune reaction occurring in most of human body and can be divided by chronic and acute types. The latter reacting to physical stimulations or foreign body infections induces tissue injury instantly and, on the other hand, the former takes longer to occur and lasts longer, involving characteristic infiltration of monocytes, macrophages, lymphocytes, and other plasma cells, inducing fibrosis or vascularization via tissue destruction and healing process [16]. Macrophages, spreading out all the body tissues, are immune cells taking charge of the innate immune responses, preying on and phagocytizing foreign bodies, bacteria, virus, and aged cells [17]. And macrophages carry out important roles in the process of inflammation by producing many inflammatory mediators, that is, proinflammatory cytokines, including interleukin-1*β* (IL-1*β*) and tumor necrosis factor-*α* (TNF-*α*), nitric oxide (NO), or prostaglandins (PG) [18]. The activated macrophages, for example, secrete a large quantity of inflammatory mediators in the primary inflammatory reaction, inducing various inflammatory diseases such as bronchitis, arthritis, multiple sclerosis, atherosclerosis, stroke, degenerative brain diseases, and viral infection, often leading to aggravation of the diseases [19, 20]. Therefore, researches to discover anti-inflammation agents effectively reducing the diverse anti-inflammatory mediators in the midst of inflammation have flourished [16]. So, the in vitro researches showing anti-inflammatory effects, using a single medicinal herb or combined extracts of multiple medicinal herbs, have been reported [6].

Examining the existing view of the medical action of Scutellariae Radix, the extract of Scutellariae Radix, or the components of Scutellariae Radix, especially the medical action of baicalein and wogonin, flavonoids, has been actively researched [8] on the subjects of antitumor and anti-inflammatory effects [5, 7–10, 21–23]. Particularly, Yoon et al. reported that water extract of Scutellariae Radix inhibits the production of NO and synthesis of IL-6 and IL-10 in the LPS-stimulated macrophages [7]; Ha and Kim reported that baicalein, a primary component of Scutellariae Radix, regulates the production of inflammatory cytokines, such as TNF-*α*, IL-1*β*, and IL-6, and the expression of COX-2 mRNA, and it represses the microglial activity, suggesting that the flavonoid could be a good candidate for the therapy of central nervous system inflammation [8]. Besides, Park also reported that the water extract of Scutellariae Radix keeps increasing or maintaining the hydrogen peroxide production in mouse macrophages, implicating immunopotentiating effect of the extract [9]. However, most of these reports are subjected to a single extract of Scutellariae Radix. The research of Kim et al. employed combined extracts including Scutellariae Radix, finding that Huanggeumjakyak-tang is effective as an anti-inflammation since the agent treated in macrophages repressed the production of NO, PGE_2_, and IL-6 and the expression of iNOS and COX-2 mRNA [5]. In this context, since Scutellariae Radix has shown to be preventive as well as having therapeutic effects in various inflammation-related diseases, the authors of this study investigated the effect of Scutellariae Radix combined with other agents on the anti-inflammatory effect.

Liriopis Tube is reported to have diverse medical actions, such as antidiabetic effect, anti-inflammation, and suppressing Ig M antibody production [11, 24–26]. Rhee et al. reported that anti-inflammatory action, in particular, of the extract of Liriopis Tube is effective in pulmonary fibrosis by inflammation adjustment via increasing macrophages ratio and decreasing lymphocytes and neutrophils ratios [11]. In addition, Lee et al. reported the effects of Liriopis Tube combined with schizandra on the levels of IL-4, IL-5, and IL-6 in an asthma animal model [25].

As yet, experiment showing the effect of anti-inflammation of the combined extracts of Scutellariae Radix and Liriopis Tube has not been reported. The water extracts of Scutellariae Radix and Liriopis Tube were combined, yielding the testing sample (SL), which was applied to RAW 264.7 cells, a mouse macrophage cell line, to investigate the effect of the combined extracts on the anti-inflammation effect by measuring cell viability, NO production, intracellular free calcium level, and production of various cytokines.

In this study, SL treatment at 25, 50, 100, and 200 *μ*g/mL did not reduce significantly the cell viability; rather it increased the rate significantly compared with those of control, implying that SL does not induce cellular toxicity on the macrophage cell line (Figure 1).

NO is known to be an inflammation modulator by involving various physiological functions such as vasodilation, neurotransmitter system, antibacteria, and immunomodulation. NO, produced as radical by NOS activity on L-arginine, plays an important intracellular secondary signal transducer. NOS can be divided into two classes, constitutive NOS (cNOS) and inducible NOS (iNOS). cNOS largely works on vasodilation and neurotransmission and NO production by cNOS has an important role in body homeostasis [27]. iNOS involves immune toxicity and is activated by a variety of stimuli, such as LPS, interferon-*γ* (IFN-*γ*), IL-1, and TNF-*α*. The enzyme generates NO for a long period of time in macrophages, vascular smooth muscle cells, endothelial cells, liver cells, and cardiac muscle cells, by which high level of NO production in vivo results in destruction of host cells, vasodilation by shock, and tissue destruction by the induced inflammation reaction [28, 29]. In this study, SL at 50 and 100 *μ*g/mL decreased the production of NO significantly. When the sample was applied to RAW 264.7 cell stimulated by LPS, SL at all the concentrations treated (25, 50, 100, and 200 *μ*g/mL) decreased significantly the production of NO (Figures 2 and 3).

Calcium is known to play a very important role in inflammation. The activated signal transduction by Toll-like receptor increases intracellular free calcium, which subsequently mediates the increase of diverse inflammatory mediators [6]. SL treatment at 25, 50, 100, and 200 *μ*g/mL suppressed significantly the intracellular free calcium production in RAW 264.7 cells stimulated by LPS (Figure 4).

Cytokines are water-soluble proteins produced in human body cells, regulating immunity and inflammation by affecting growth, differentiation, expansion, and activation of immune cells. IL-6 has an important role in the host defense mechanism and immune response [30]. SL treatment at 25, 50, and 100 *μ*g/mL suppressed significantly the IL-6 production in RAW 264.7 cells stimulated by LPS (Table 1).

MIP is a member of the chemokine subfamily that was originally purified from the conditioned media of an LPS-stimulated macrophage cell line. Two major forms of MIP, MIP-1*α* and MIP-1*β*, are highly related in immune and inflammatory response. They activate granulocytes which can lead to acute neutrophilic inflammation. And they also induce the synthesis and the release of other proinflammatory cytokines such as IL-1, IL-6, and TNF-*α* from fibroblasts and macrophages. MIP-1 is best known for its chemotactic and proinflammatory effects but can also promote hematopoiesis. Particularly, MIP-1*β* is expressed by T cells, B cells, and monocytes after antigen or mitogen stimulation [31, 32]. SL treatment at 25, 50, and 100 *μ*g/mL suppressed significantly the MIP-1*α*, the MIP-1*β*, and the MIP-2 production in RAW 264.7 cells stimulated by LPS (Table 1).

CSF, facilitating growth and differentiation of bone marrow stem cells as a hematopoietic growth factor, has gotten much attention since the factor has been reported to facilitate the differentiation of granulocytes and macrophages. Granulocyte colony-stimulating factor (G-CSF), macrophage colony-stimulating factor (M-CSF), and granulocyte macrophage colony-stimulating factor (GM-CSF) belong to CSF and multi-colony-stimulating factor is also known as IL-3 [5, 33–35].

G-CSF facilitates granulopoiesis and its level is increased in respiratory disease such as asthma [30, 31]. SL treatment at 25, 50, and 100 *μ*g/mL suppressed significantly the G-CSF production in RAW 264.7 cells stimulated by LPS (Table 1).

M-CSF produced in osteoblasts or mesenchymal stem cells involves the differentiation of osteoclasts and big mononuclear cells and has an important role in the recombination of monocyte-macrophage cell lines [6]. SL treatment suppressed M-CSF production in RAW 264.7 cells stimulated by LPS but not significantly (Table 1).

GM-CSF facilitates growth and activation of granulocytes and macrophages as a protective tool against infection and inflammation [35]. SL treatment suppressed GM-CSF production in RAW 264.7 cells stimulated by LPS but not significantly (Table 1).

The role of TNF-*α* is in the regulation of immune cells. TNF-*α* can induce the release of chemokines, prostaglandins, protease, and growth factors by activating endothelial cell, neutrophil, B cell, and so forth [16]. SL, just treatment at 25 *μ*g/mL, suppressed significantly the TNF-*α* production in RAW 264.7 cells stimulated by LPS (Table 1).

LIF is an IL-6 class cytokine and RANTES are known for kinds of chemokine. SL treatment suppressed LIF and RANTES production in RAW 264.7 cells stimulated by LPS but both not significant (Table 1).

These results suggest that SL treatment does not harm the viability of cells and SL could be used to alleviate the symptoms of acute and chronic inflammatory diseases which are induced by overproduction of various inflammatory mediators in macrophages, for example, by infectious agents such as LPS, or inflammatory autoimmune diseases [36, 37]. Further study, for example, the effect of SL on more diverse inflammatory mediators, is demanded for the follow-up research to find out the possibility of SL as a preventive and therapeutic medicine for various inflammatory diseases.

## 5. Conclusions

The water extracts of Scutellariae Radix and Liriopis Tube were combined, yielding the testing sample (SL), which was applied to RAW 264.7 cells to investigate the effect of SL on the cell viability, NO production, intracellular free calcium level, and production of various cytokines. As described in this study, SL treatment at all the concentrations of 25, 50, 100, and 200 *μ*g/mL did not show any particular toxicity in the mouse macrophages; rather it increased cell viability and at 50 and 100 *μ*g/mL NO production was decreased significantly. In addition, SL at all the concentrations of treatment significantly suppressed the production of NO and the increase of the intracellular free calcium in the mouse macrophages stimulated by LPS. The increases of IL-6, MIP-1*α*, MIP-1*β*, MIP-2, and G-CSF induced by LPS treatment in mouse macrophages were significantly suppressed by the treatment of SL at the concentrations of 25, 50, and 100 *μ*g/mL.

These results implicate that SL has anti-inflammatory effect by suppressing the production of various inflammatory mediators in macrophages.

## Figures and Tables

**Table 1:** Effects of SL on various cytokines production in LPS-treated RAW 264.7 cells.

Cytokines (pg/mL)	Normal	Control	SL25	SL50	SL100
IL-6	58.17 ± 4.54	25515.17 ± 96.13^#^	24079.17 ± 632.48^*∗*^	23759.67 ± 243.89^*∗∗*^	24204.33 ± 614.87^*∗*^
MIP-1*α*	7259.00 ± 614.07	27791.33 ± 33.50^#^	25886.33 ± 247.03^*∗∗*^	25939.67 ± 58.29^*∗∗*^	26115.17 ± 381.14^*∗∗*^
MIP-1*β*	7369.17 ± 2454.09	2588.00 ± 352.36^#^	24379.50 ± 287.76^*∗∗*^	23904.33 ± 386.25^*∗∗*^	24090.33 ± 276.86^*∗*^
MIP-2	64.33 ± 17.04	25599.83 ± 68.31^#^	24114.50 ± 494.84^*∗∗*^	23695.67 ± 163.49^*∗∗*^	24334.00 ± 132.53^*∗*^
G-CSF	84.33 ± 32.13	27370.00 ± 7.00^#^	25154.17 ± 341.76^*∗∗*^	24702.50 ± 503.57^*∗∗*^	25769.00 ± 132.32^*∗∗*^
M-CSF	40.67 ± 4.62	77.00 ± 6.08^#^	68.67 ± 7.37	69.00 ± 5.29	64.17 ± 6.75
GM-CSF	57.33 ± 3.79	6185.17 ± 463.36^#^	4947.83 ± 706.00	4781.00 ± 547.75	6210.67 ± 1406.94
TNF-*α*	169.33 ± 17.50	7013.00 ± 245.35^#^	6393.67 ± 269.24^*∗*^	6561.17 ± 656.97	6685.50 ± 1020.25
LIF	38.50 ± 4.92	7757.33 ± 376.11^#^	6878.83 ± 644.82	6626.33 ± 615.58	7765.50 ± 794.14
RANTES	134.67 ± 19.01	13871.83 ± 257.82^#^	13535.33 ± 1275.87	13082.50 ± 907.42	13789.67 ± 420.85

Normal: cytokine production in the RAW 264.7 cells treated with media only.

Control: cytokine production in the RAW 264.7 cells treated with LPS (1 *μ*g/mL).

SL25: cytokine production in the LPS-stimulated RAW 264.7 cells incubated with Scutellariae Radix and Liriopis Tuber water extract at the concentration of 25 *μ*g/mL.

SL50: the production of cytokines in the LPS-stimulated RAW 264.7 cells incubated with Scutellariae Radix and Liriopis Tuber water extract at the concentration of 50 *μ*g/mL.

SL100: the production of cytokines in the LPS-stimulated RAW 264.7 cells incubated with Scutellariae Radix and Liriopis Tuber water extract at the concentration of 100 *μ*g/mL.

Results are represented as mean ± SD.

# represents *P* < 0.05 compared to the normal.

*∗* represents *P* < 0.05 compared to the control.

*∗∗* represents *P* < 0.01 compared to the control.
